# Opportunities and strategies for microsurgery training as a resident: Reflections and recommendations

**DOI:** 10.1016/j.jpra.2025.10.009

**Published:** 2025-10-17

**Authors:** Hatan Mortada

**Affiliations:** a Division of Plastic Surgery, Department of Surgery, King Saud University Medical City, King Saud University, Riyadh, Saudi Arabia; b Department of Plastic Surgery & Burn Unit, King Saud Medical City, Riyadh, Saudi Arabia

**Keywords:** Microsurgery, Surgical training, Residency education, Operating room opportunities, Simulation training, Mentorship in surgery

## Abstract

Microsurgery training presents significant challenges for residents, who must navigate the dual demands of acquiring advanced technical skills and securing sufficient operating room (OR) exposure. This commentary draws on my personal experience as a plastic surgery resident with a keen interest in microsurgery to offer practical insights and recommendations. I discuss the critical role of simulation-based practice, the importance of mentorship and professional networking, and strategies for advocating OR participation. These approaches have been instrumental in my own journey and are intended to guide residents seeking excellence in this demanding yet rewarding field. By fostering a proactive and systematic approach to training, we can bridge the gap between skill acquisition and clinical application.

## Introduction

Microsurgery is a core skill in reconstructive surgery, with a steep learning curve that demands precision and repeated practice under supervision.^1^^,^^2^

The shift towards reduced resident work hours and increased patient safety protocols has limited opportunities for hands-on training in the operating room.^3^ This constraint underscores the need for alternative training methods, such as simulation-based learning and structured curricula, to ensure residents achieve competence.^4^

As a final-year plastic surgery resident in the Saudi Board of Plastic Surgery (SB-PLAST) with a focus on reconstructive microsurgery, I have pursued diverse strategies to enhance my training. This article shares practical reflections drawn from five years of clinical experience, structured local education, and international observerships. Supported by relevant literature, these insights aim to guide residents and educators in advancing microsurgical education.

## Simulation-based practice

**Figure 1:**
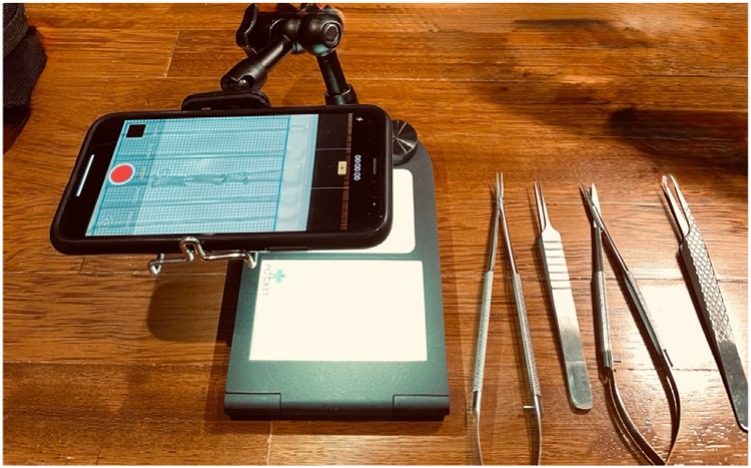
The Pocket Microsurgery Trainer™. A portable training device allowing residents to practice microsurgical techniques anywhere using their smartphone or tablet. The trainer includes replaceable microvascular training cards for performing end-to-end, end-to-side, and side-to-side anastomoses.

During my residency, I dedicated significant time to simulation-based microsurgical practice, which allowed me to develop and refine fundamental and intermediate skills such as suturing, vessel handling, and performing microvascular anastomoses. Devices like the Pocket Microsurgery Trainer™ and synthetic microvessels (Figure 1) offer practical, portable simulation for real-life microsurgical scenarios. To maximize the benefits of simulation, I recommend: (1) Structured Simulation Programs: Residency programs should incorporate mandatory and progressive simulation sessions, culminating in advanced scenarios. (2) Objective Feedback: Regular feedback from faculty or peers during simulation exercises enhances skill acquisition. Video recording and self-assessment further contribute to improvement. (3) Integration with OR Practice: Simulation is most effective when integrated with OR exposure, enabling residents to apply learned techniques in real surgical contexts. Several digital platforms complement hands-on simulation. For instance, Touch Surgery (by Medtronic) offers free modules that simulate various microsurgical procedures through interactive animations. In contrast, SimuSurg and UpSurgeOn provide a hybrid model—free access to select content with premium modules requiring subscription. Some platforms, such as the Osso VR simulator, target institutional use and may require institutional licenses. These tools can reinforce anatomic understanding and procedural steps, particularly for early learners.

## Mentorship and networking

**Figure 2:**
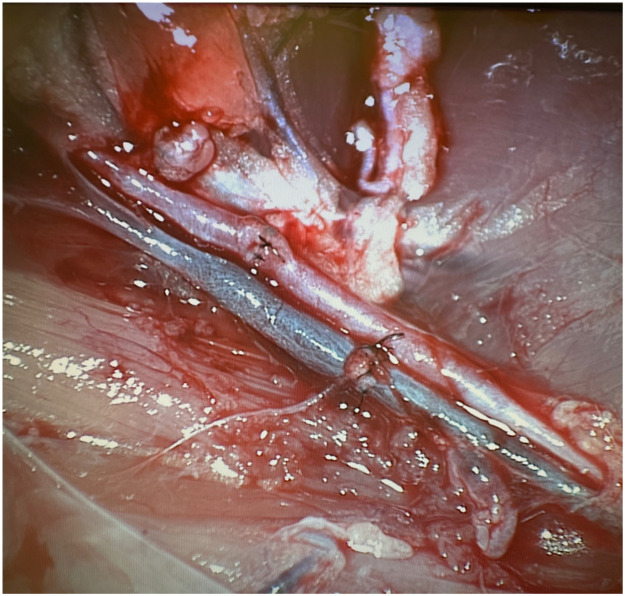
End-to-end femoral artery and vein anastomoses performed by the author during the basic advanced microsurgical skills training course at Columbia University, New York, USA. The training course focused on refining vascular anastomosis techniques under the guidance of Dr. Yelena Akelina.

Mentorship has been a pivotal factor in my microsurgery training. By seeking guidance from skilled microsurgeons, I gained technical feedback and insights into case selection, operative planning, and career development. Regular discussions and feedback sessions with mentors helped me identify areas for improvement and strategies for overcoming challenges. Additionally, professional networking through local and international societies (e.g., the International Microsurgery Club) expanded my exposure to innovations and emerging trends. Attending workshops, symposia, and hands-on microsurgery courses, such as the Basic Advanced Microsurgical Skills Training Program at Columbia University Irving Medical Center, further refined my skills (Figure 2). Visiting mentors has also been instrumental in my development. I had the privilege of enhancing my microsurgical skills under the guidance of renowned experts, including Prof. JP Hong at the Asan Medical Center in South Korea, and Prof. Sabapathy at Ganga Hospital, India, during the 2023 Operative Hand Course, where I gained invaluable insights and hands-on experience in advanced microsurgical techniques.^5^, ^6^ These experiences allowed me to observe advanced microsurgical techniques firsthand and integrate novel strategies into my practice. Spending weeks in these world-class institutions helped me understand complex reconstructions and improve my confidence in managing challenging cases. Residents interested in more immersive training may benefit from international fellowships. Examples include the Ganga Microsurgery Fellowship (India), Chang Gung Memorial Hospital Microsurgery Fellowship (Taiwan), and MD Anderson’s Reconstructive Microsurgery Fellowship (USA). Some programs, such as the EURAPS Research Council Visiting Fellowship, offer funding support for short observerships in top European units. These fellowships not only enhance technical expertise but also broaden academic and cultural perspectives. Key actionable strategies for residents seeking microsurgical training are summarized in Table 1.

**Table 1:** Practical recommendations for residents in microsurgery training.

Strategy	Description
Identify mentors	Seek mentors with strong microsurgical expertise and a commitment to resident education.
Engage in societies	Join local and international microsurgery societies to access educational events and networking.
Learn beyond the OR	Attend courses, webinars, and international symposia to complement hands-on training.
Shadow experts	Visit high-volume microsurgical centers to observe techniques and refine operative skills.

## Proactive involvement in the operating room

Securing OR opportunities as a resident can be challenging, especially in competitive training programs. To overcome this, I adopted a proactive approach: demonstrating readiness, preparing meticulously for cases, and consistently volunteering for microsurgical procedures.

Specific strategies that worked for me include: (1) Case Preparation: Reviewing anatomy, studying operative steps, and understanding potential complications for each case fostered trust among attending surgeons. (2) Building Relationships: By consistently showing interest and reliability, I established rapport with senior residents and attendings who advocated for my inclusion in cases. (3) Progressive Responsibility: Starting with assisting roles and progressing to supervised hands-on tasks ensured I developed skills incrementally while maintaining patient safety.

## Mentor’s perspective

A senior microsurgeon consultant noted that effective practical training requires initiative, persistence, and self-directed practice. The described approach—utilizing simulation, early preparation, and observerships—represents a resident-driven model aligned with contemporary educational goals, particularly when supported by institutional mentorship.

## Conclusion

Microsurgery training demands a proactive, structured approach. Simulation, mentorship, networking, and OR engagement form the foundation of skill development. Despite challenges, resident-driven learning and supportive training environments can help trainees achieve clinical competence and prepare for independent practice.

## Funding

No funding was received.

## Supplementary materials

Supplementary material associated with this article can be sent upon reasonable request from the corresponding author.

## Ethical approval

Ethical approval was waived due to the nature of the study.

## CRediT authorship contribution statement

**Hatan Mortada:** Conceptualization, Investigation, Software, Writing – review & editing.

## Declaration of competing interest

The author(s) declare no potential competing of interest with respect to the research, authorship and/or publication of this article.
